# New Method Application for Marker-Trait Association Studies in Plants: Partial Least Square Regression Aids Detection of Simultaneous Correlations

**DOI:** 10.19080/ARTOAJ.2017.12.555864

**Published:** 2017-12-15

**Authors:** Eliane Thaines Bodah, Bruce Weir

**Affiliations:** Department of Biostatistics, University of Washington, USA

**Keywords:** PLSR, Multivariate analyses, Marker-trait association, Plant methods

## Abstract

In this work, we investigated the suitability of performing partial least square regression (PLSR) on genotype-phenotype datasets to identify marker-trait associations. We utilized data collected on a cotton (*Gossypium hirsutum L*.) recombinant inbred line (RIL) mapping population that was evaluated under contrasting irrigation treatments, well-watered and water-limited conditions, in a hot, arid environment in 2012. Two phenotypic data sets were used in combination with the genetic data which consisted of 841 marker loci assigned to 117 linkage groups. The first dataset contained canopy traits that were gathered using a mobile, high-throughput phenotyping platform and included canopy temperature (CT), normalized difference vegetation index (NDVI), and canopy height (CHT) with leaf area index (LAI) being derived from NDVI and CHT measurements. The second phenotypic data set consisted of 14 elemental concentration measurements corresponding to the following elements: P, K, Ca, Mn, Fe, Zn, Ni, Cu, As, Co, Rb, Mo, S, and Mg. To conduct the PSLR analyses we used the “pls” and “pls depot” available in R statistical software version 3.2.4. The PLSR bi plot from the analysis of the first dataset showed that three (LAI, NDVI, and CHT) out of the four canopy traits were highly correlated, and by using multivariate analysis of variance (MANOVA), we detected 22 significant (p<0.01) marker-trait associations for the four traits. In contrast to the canopy trait analysis, our PLSR bi plot for the second dataset showed varying correlations for each of the 14 traits. Because of the lack of distinct trait similarities, MANOVA was not an ideal option to test for marker-trait associations so we implemented a jackknife re sampling technique. Jackknife re sampling failed to detect significant marker effects for several of the 14 elemental concentration traits. Thus, our future work aims to test other re sampling techniques such as boot straping for traits that do not exhibit high correlation. Overall, PLSR was a very informative way to comprehend data structure, displaying correlations within markers, within traits, and between marker and traits in one bi plot. Further studies are still needed to leverage detection of additional variance in correlated datasets and to prevent spurious results. To the best of our knowledge, this is the first time PLSR has been reported in such a context.

## Introduction

Although many quantitative traits that are measured on populations exhibit moderate to high correlations, standard marker-trait association methods are univariate and thus unable to capitalize on this trait relatedness. A common approach to handling this situation so that standard univariate approaches can be used is to create a composite score that combines the measurements of multiple traits into one. However, the analysis of these composite scores is known to entail loss of statistical power [1].

Aiming to improve complex marker-trait association studies and to contribute to a better understanding of correlated datasets, we performed partial least square regression (PLSR) in two drought-related cotton phenotypic datasets. To the best of our knowledge, this is the first time PLSR has been reported in such context.

PLSR was first proposed to solve the multi collinearity problem in regression or calibration [2,3]. In this approach, predictors (X) are described by a principal components type of model, combined with a regression relation between the X scores (component or factor matrix) and the response variable (Y), also referred as response matrix [2,4].

In PLSR, X and Y are projected into new spaces. The projection matrices are named T and U, respectively. P and Q are the orthogonal loading matrices for X and Y, respectively [4]. Through simultaneous decomposition of X and Y, PLSR aims to explain as much as possible of the variance between predictors and response variables [5].

In our work, we used markers as predictors, X, and traits as the responses variable, Y, to find the multidimensional direction in the X space that explained the maximum multidimensional variance direction in the Y space. Through the construction of a bi plot, correlations within markers, within traits, and between markers and traits, can all be detected and displayed [6].

## Methods

### Datasets

PLSR was performed on two cotton datasets that were collected during 2012 at the University of Arizona’s Maricopa Agricultural Center (MAC) in Maricopa, AZ. Phenotypic data were collected on 95 recombinant inbred lines (RILs) from the TM-1×NM24016 bi parental mapping population [7,8] evaluated under contrasting irrigation regimes, well-watered and water-limited conditions. This population has been previously genotyped with 841 molecular markers which are assigned to 117 linkage groups.

The first dataset analyzed contained three traits that were directly measured on the cotton canopy using a mobile, high-throughput phenotyping platform which has been previously described [9]. These canopy traits were canopy temperature (CT), normalized difference vegetation index (NDVI) and canopy height. Using the data for NDVI and CHT, leaf area index (LAI) was derived according to [10] with parameter estimates specific to Southwestern Arizona obtained previously [11]. Canopy data were collected twice per day, at 10 am and 1 pm, several times throughout the growing season starting at flowering and going through plant maturity.

The second dataset analyzed for this work is a set of 14 elemental concentrations measured on the seeds of the RIL population evaluated in 2012. The elements assayed for this work include P, K, Ca, Mn, Fe, Zn, Ni, Cu, As, Co, Rb, Mo, S, and Mg. All the data collection and univariate association tests were conducted and published by [12] or are under review.

### Data Processing and Analyses

Data processing of the phenotypic measurements was performed in both datasets using a differential between the well-watered and the water-limited treatments. The idea of a trait differential was to have only one measurement for each of the traits analyzed to avoid having to conduct separated multivariate analyses for each irrigation treatment. For instance, if a plant reaches a canopy height of 10 cm if well-watered, and of 6 cm if water-limited due to drought-stress, we would use the difference in growth of 4 cm as the differential phenotype in Y.

Marker-trait association studies in plants are generally conducted using quantitative trait loci (QTL) analyses. QTL software that are currently available can use multiple markers, however they can only use one trait at the time. Our goal was to use all the traits and all the markers in one analysis.

In this work, we propose using PLSR as a multivariate tool due to the simultaneous correlations input and the increase power. Further more, its bi plot offers a great graphical display of all the data to aid researchers in decision-making processes. For the first dataset with four drought-related traits we performed PLSR as well as a principal component analysis (PCA) followed by multiple analysis of variance (MANOVA).

PCA extracted the dominant patterns in the trait matrix in terms of a complementary set of scores and loading plots [13], by replacing the p original variables by a smaller number, q, of derived variables [14]. The first few principal components were integrated into a MANOVA to detect significant marker effects on the four traits. Markers were then annotated using the information available at a publicly available cotton dataset (https://www.cottongen.org/species/Gossypium_hirsutum/nbi-AD1_genome_v1.1).

For the second dataset, we also performed PLSR but not MANOVA. MANOVA generally requires further discriminant analyses or univariate comparisons, which are still under preparation by [12]. To estimate variances, we then followed PLSR with jackknife re sampling technique. Jackknife computes statistics on n separate samples of size n-1, where each sample is the original data with a single observation omitted [15].

All statistical analyses were performed using R statistical software version 3.2.4. [16], mainly employing the R packages *pls* and *pls depot* packages [16].

## Results

### Identifying markers associated with CT, NDVI, CH and LAI

In the first dataset, we performed PLSR, PCA and MANOVA to detect associations between markers and the canopy traits CT, NDVI, CHT, and LAI.

**Figure 1:**
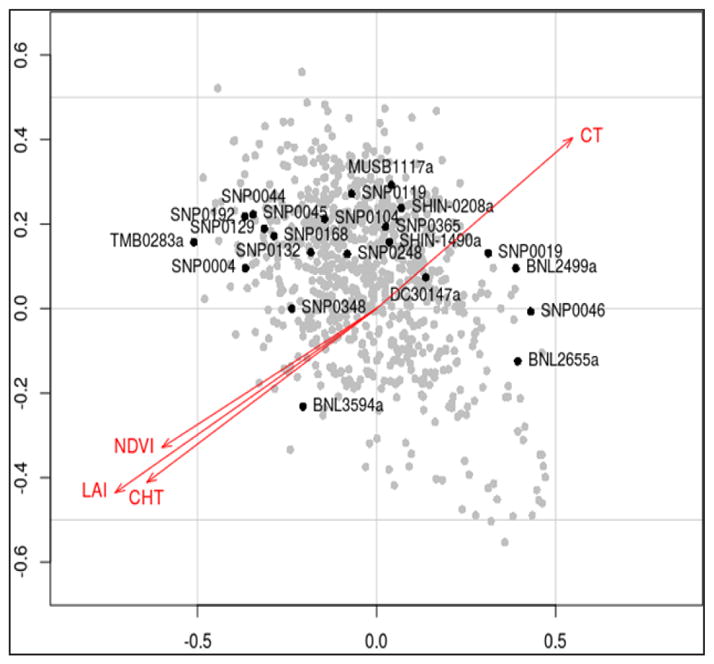
Partial Least Square Regression: correlation biplot; showing trait components in red, and marker components in gray and black (significantly associated markers). Within traits, negative correlations for LAI, NDVI, CHT, and positive correlation for CT were detected.

Through PLSR it was possible to detect correlations within markers, within traits, and between markers and traits as demonstrated by the biplot (Figure 1). The biplot shows that latent vectors for NDVI, LAI and CHT presented high correlation among them. However, the CT vector was shown in the opposite direction of the other traits, suggesting an inverse relationship between CT and the other three traits.

Partial correlation coefficients were then extracted for the traits (Table 1). Negative correlations were detected for most trait responses when looking at the latent vectors t_1_, t_2_, t_3_. For instance, in t1 the coefficients were −0.72 for LAI, −0.61 for NDVI, and −0,63 for CT. A positive correlation was detected for CHT with a respective coefficient of 0.56 (Table 1).

Partial correlation within markers, and between markers and trait were difficult to observe in the biplot due to the high number of markers in one plot (841). Statistical significance was also difficult to find just based on the regression coefficients obtained from the PLSR. An efficient multivariate method to detect association is a MANOVA.

**Figure 2:**
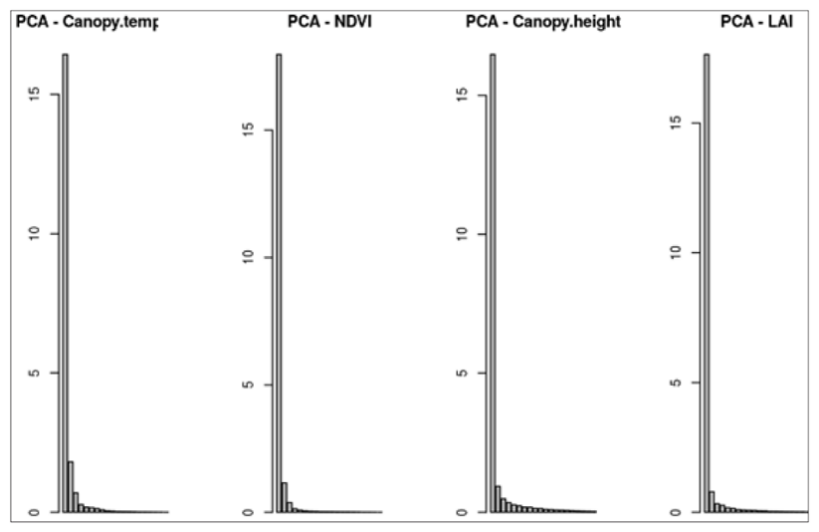
Principal Component Analysis (PCA) results for each drought-related trait; showing variation within each component, from left to right: canopy temperature, NDVI, canopy height, and LAI. Y-axis is percent variation explained by PC on X-axis.

To reduce the dimensionality of this complex dataset without the use of an index, we first needed to extract the principal components (PC) of the trait measurements. The first few principal components captured the major data variation and were then used for the MANOVA (Figure 2).

**Figure 3:**
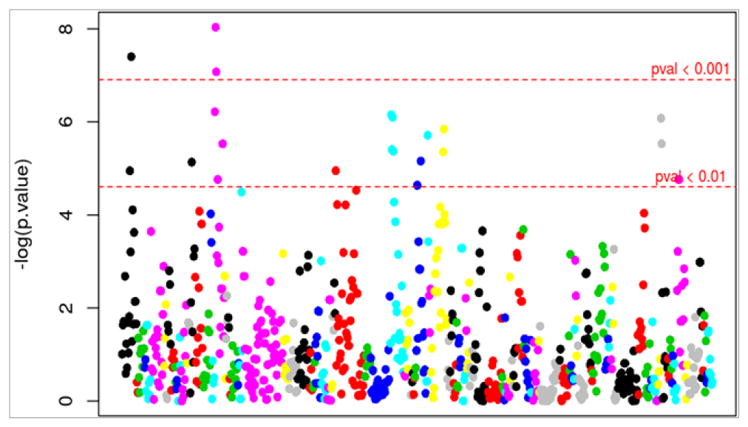
Markers associated with drought identified using Principal Component Analysis followed by Multivariate Analysis of Variance in different linkage groups (color-coded). The Y axis displays the −log(p-value); where −log (0.01) ~ 4.6. A total of 22 significant associations were detected at p<0.01 based on the Pillai-Bartlett Trace test.

After performing MANOVA, we detected 22 markers that were significantly associated (p<0.01) with drought resistance/tolerance in plants based on the Pillai-Bartlett Trace test [17] (Figure 3).

Significant markers were then annotated from the previously reported cotton dataset (Table 2). Successfully annotated markers with their respective functions are listed as follows: BNL3594a -uncharacterized protein, MUSB1117a- ribosomal protein S25 family protein, SHIN-1490a- predicted protein, SNP0365- ubiquinol-cytochrome C reductase hinge protein, SNP0119- putative uncharacterized protein, SNP0132-neurochondrin family protein, SNP0168- topless-related protein, SNP0129- tesmin/TSO1-like CXC domain-containing protein, SNP0004-DDB1-binding WD40 protein hypersensitive to ABA 1, SNP0348- NAC domain-containing protein, and SNP001 -ABA receptor PYL8 OS.

### Identifying markers associated with elemental traits

The second dataset contained the same 841 markers used in the first set of analyses. However, instead of looking at four drought related traits, we have 14 elemental traits P, K, Ca, Mn, Fe, Zn, Ni, Cu, As, Co, Rb, Mo, S, and Mg. For these analyses, we performed PLSR followed by jackknife.

The biplot originated from the PLSR was very informative, showing that some traits were more correlated than others (Figure 3).

For instance, latent vectors for Ni and Zn shows a high correlation as they are very close in the graph. In the same area, there is Fe, Cu, and Ca that can be said to have correlation with Ni and Zn. Another correlated set includes Rb, Mo, S and P. Mg is standing alone in opposition to Zn. In addition, the following ions also stand alone: Co, K, and As.

Partial correlation coefficients within the traits were then extracted (Table 3). Considering the first latent vector (t1), positive correlations were detected for P, K, Mo, S, As, Mg, Co, Rb; while negative correlations were detected for Ca, Mn, Fe, Zn, Ni, and Cu. With such a variety of results it would not be appropriate to proceed with a MANOVA, as some of the traits are totally independent of others.

In order to detect statistical significance for the marker effect on the trait we performedjackknife resampling. Then, we were able to list the most singificant markers for P, K, Ca, Mn, Mo, and Rb (Table 4). The resampling failed to detect any significant markers for Fe, Zn, Ni, Cu, As, Co, S, and Mg.

## Discussion

In this work, we proposed the use of multivariate approaches to detect marker-trait associations in two cotton databases. For both, PLSR was effective as an exploratory method, especially to display correlation within traits.

We have also observed that from the drought-related traits LAI, NDVI, and CHT are highly correlated. This finding suggests that LAI, NDVI and CHT can be combined in analyses as reliable drought indicators. CT was inversely correlated to the other three traits in our study. Even though we did not find correlation between CT and the other drought-related traits, there is evidence that CT relates to yield reductions associated with high temperature events [18].

Pearson’s pair wise correlation coefficients tables are standard in plant research reports. Such tables can have massive information. Our PLSR bi plot can aid researchers to explore all their data, and visualize correlations in a more informative way.

Conducting a MANOVA in addition to the PLSR, enabled the identification of 22 important markers for candidate drought related genes. After annotation, some important markers were found to be related to the 5- ubiquinol-cytochrome C reductase hinge protein, topless-related protein, tesmin/TSO1-like CXC domain-containing protein, DDB1-binding WD40 protein hypersensitive to ABA 1, NAC domain-containing protein, and the ABA receptor PYL8 OS.

Ubiquinol-cytochrome C reductase hinge protein is involved in mitochondrial electron transport and it is in the mitochondrial respiratory chain complex III [19]. The topless-related protein may activate TIR-NB-LRR R protein-mediated immune responses through repression of negative regulators such as CNGC2/DND1 (PubMed: 20647385). It is also a negative regulator of jasmonate responses [20]. Tesmin/TSO1-like CXC domain-containing protein plays a role in development reproductive tissues [21].

Marker annotation was important to elucidate how drought is affecting plant development as well as to find lines that might have some tolerance to this a biotic stress. The annotation also confirmed that our analyses were useful, as they could detect markers associated with the drought-related traits. An example is the ABA markers DDB1-binding WD40 protein hypersensitive to ABA 1 and the ABA receptor PYL8 OS. The degree of biosynthesis and accumulation of ABA in a crop cultivar is a possible indicator of drought tolerance [22]. In addition, NAC domain-containing protein was characterized in response to water deprivation [23].

From the significant marker detected in this work, SNP013222 (a neurochondrin family protein) on linkage group 22, and MUSB1117a (a ribosomal protein S25 family protein) on linkage group 45 were also detected by a univariate quantitative trait local (QTL) inclusive composite interval mapping (ICIM) approach performed by [12].

For the second dataset with 14 elemental traits (P, K, Ca, Mn, Fe, Zn, Ni, Cu, As, Co, Rb, Mo, S, and Mg) we also conducted PLSR. The biplot with correlation among traits was very informative, showing highly correlated traits, inversely correlated traits and traits that were independent of the others.

PLSR correlation coefficients within traits, within markers, and between markers and traits could be extracted from the analysis to give estimates of the marker effect on each trait.

However, as mentioned previously, to find significant marker-trait associations, we applied a jackknife resampling technique. The newest updates on the pls R package became available on December of 2016 and included a jackknife option to perform approximate t-tests of regression coefficients based on jackknife variance estimates. In the literature, bootstrapping is also cited as an applicable resampling technique.

The resulting p-values after performing jackknife should not be used uncritically, and should perhaps be regarded as mere indicator of (non) significance. This to the fact that the distribution of the regression coefficient estimates and the jackknife variance estimates are unknown (at least in PLSR/PCR) [24].

## Conclusion

PLSR is a very informative way to help comprehend how data are structured, displaying correlations within markers, within traits, and between marker and traits in one biplot.

For the first dataset, PCA followed by MANOVA seemed to be an effective approach to detect association. However, further discriminant analyses might be needed if univariate analyses are not available. Furthermore, our first dataset univariate analyses were published by [7].

PLSR offers partial correlation coefficients and resampling techniques are necessary to estimate variances. In this work, we used jackknife resampling but it failed to detect significant marker effects for several traits. This fact could also be due to no real effects of markers on the studied traits.

Future work will include performing PLSR in other crops as well as looking at bootstrap as an alternative resampling technique. Overall, multivariate approaches in marker-trait association studies still need to be improved and broadly tested to leverage detection of additional variance and prevent spurious results.

## Figures and Tables

**Figure 4:**
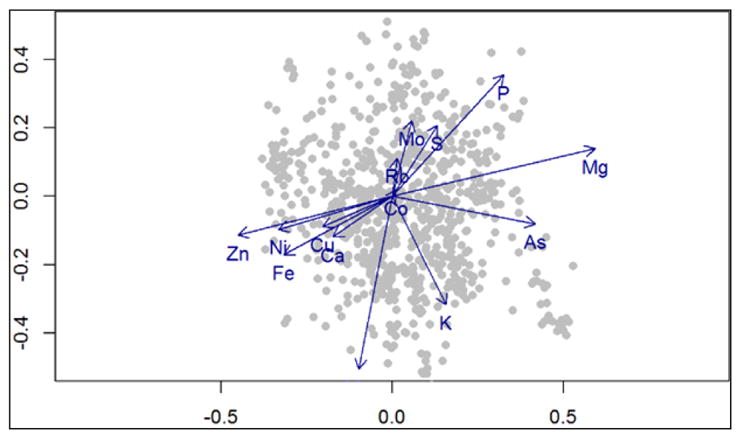
Partial Least Square Regression correlation biplot; plot showing trait components in blue, and markers in gray. Proximity in the graph, such as in the vectors for Ni and Zn, shows that the components are highly correlated. Opposing direction for vectors such as in Zn and Mg, shows that the traits are inversely correlated.

**Table 1:** Partial correlation coefficients between Y (CT, NDVI, CHT, LAI) and Xj were observed for each latent vector of matrix T (t^1^, t^2^, and t^3^).

Trait	T_1_	T_2_	T_3_
CT	0.56	0.28	0.48
NDVI	−0.61	−0.21	−0.5
CHT	−0.63	−0.42	−0.13
LAI	−0.72	−0.42	−0.22

**Table 2:** Significantly drought-associated markers at p<0.01; showing marker ID, linkage group (LG), and trait coefficients for canopy height (CHT), normalized 50 difference vegetation index (NDVI), canopy temperature (CT), and leaf area index (LAI).

Marker ID	LG	CHT	NDVI	CT	LAI
TMB0283a	1	−0.47	0.82	1.24	1.37
SNP0192	1	−0.71	1.03	1.62	1.73
BNL3594a	17	−0.75	0.75	0.59	0.97
SNP0132	22	−1.06	1.31	1.04	1.53
SNP0168	22	−1.3	1.95	1.31	1.92
SNP0129	22	−0.77	1.09	0.64	1.12
SNP0044	22	−0.46	0.7	0.92	1.21
SNP0004	22	−0.53	0.85	1.19	1.44
DC30147a	42	0.41	−1.13	0.42	0.019
MUSB1117a	45	−0.13	0.09	−0.57	−0.72
SHIN-0208a	45	−0.05	−0.05	−0.6	−0.77
SHIN-1490a	45	−0.31	0.14	−0.44	−0.58
SNP0365	45	−0.56	0.36	−0.41	−0.49
SNP0348	52	−0.79	0.75	1.4	1.6
SNP0248	52	−0.95	0.75	1.73	1.91
SNP0019	53	0.25	−0.58	−1.49	−1.47
SNP0119	55	−0.9	1.12	−0.39	−0.11
SNP0104	55	−0.52	1.13	−0.65	−0.43
BNL2655a	104	2.44	−2.6	−1.17	−1.3
BNL2499a	104	2.052	−2.36	−1.14	−1.33
SNP0046	110	0.46	−0.7	−0.92	−1.21
SNP0045	110	−0.46	0.7	0.92	1.21

**Table 3:** Partial correlation between Y (P, K, Ca, Mn, Fe, Zn, Mo, S, Ni, Cu, As, Mg, Co, and Rb) and Xj were observed for each latent vector of matrix T (t_1_, t_2_, and t_3_).

Ion	t_1_	t_2_	t_3_
P	0.32599596	0.35472220	0.333397
K	0.1577072	−0.31668904	−0.30585
Ca	−0.17344796	−0.11929882	−0.33944
Mn	−0.09707526	−0.50462247	−0.20505
Fe	−0.31679096	−0.17088226	−0.02917
Zn	−0.45062098	−0.11295626	0.016705
Mo	0.0563702	0.22072761	−0.38464
S	0.13152467	0.20619102	−0.24154
Ni	−0.33292305	−0.09658496	−0.00291
Cu	−0.20325922	−0.0886891	−0.17913
As	0.41771351	−0.08055791	−0.08282
Mg	0.59218889	0.13912230	0.004347
Co	0.01165428	0.0185707	0.268
Rb	0.01356858	0.11154640	0.32252

**Table 4:** Significant markers effect on the traits (for traits P, K, Ca, Mn, Mo, and Rb), showing marker name, estimate, standard error, DF, t value, p-value, and regression coefficient.

SHIN.0473a	−70.04	35.21	94	−1.99	0.04*	2.64E+00
DPL0755a	−38.1	18.3	94	−2.08	0.04*	−1.00E-02
SNP0002	67.2	27.47	94	2.45	0.02*	8.70E-03
DPL1550a	104.18	44.11	94	2.36	0.02*	2.08E-02
SNP0126	85.28	40.29	94	2.12	0.04*	3.76E+00
SNP0138	81.29	34.82	94	2.33	0.02*	1.32E-02
SNP0043	−103.45	46.35	94	−2.23	0.03*	4.28E-03
SNP0188	90.93	41.75	94	2.18	0.03*	−5.78E-03
SNP0452	−81.38	37.49	94	−2.17	0.03*	4.51E-03
SNP0108	−76.29	36.3	94	−2.1	0.04*	4.13E-03
SNP0031	−98.13	46.4	94	−2.11	0.04*	3.71E-03
SNP0067	−98.13	46.4	94	−2.11	0.04*	3.71E-03
P-associated Marker Est. Std. Error DF t value P(>|t|) Reg. Coef.						
SNP0236	−49.13	23.8105	94	−2.06	0.04*	9.813208e-
SNP0024	34.9	17.2903	94	2.02	0.04*	−1.811791e-
SNP0163	45.31	19.7703	94	2.29	0.02*	−1.920897e-
DPL1154b	42.33	19.5603	94	2.16	0.03*	−1.984356e-
DPL0750a	−55.43	25.84	94	−2.14	0.03*	5.696450e-
DPL1846a	44.2	21.94	94	2.01	0.04*	4.15E-03
K-associateds						
Ca-associated						
SNP0173	−47	22.38	94	−2.1	0.04*	−2.83E-03
Mn-associated						
SNP0470	0.24	0.12	94	2.04	0.04*	2.33E-03
DPL1144a	0.26	0.13	94	2.04	0.04*	−2.472913075
Mo-associated						
SNP0470	−16.3	8.2	94	−1.99	0.04*	−3.74E-04
DPL1144a	−17.85	8.63	94	−2.07	0.04*	−3.78E-04
Rb-associated						
DPL1550a	3.62E-02	1.63E-02	94	2.21	0.03*	1.59E-02
CM0007a	2.37E-02	1.15E-02	94	2.05	0.04*	1.21E-02
BNL1414a	2.40E-02	1.16E-02	94	2.06	0.04*	7.14E-03
